# Self-reported HIV-positive status but subsequent HIV-negative test result using rapid diagnostic testing algorithms among seven sub-Saharan African military populations

**DOI:** 10.1371/journal.pone.0180796

**Published:** 2017-07-07

**Authors:** Judith Harbertson, Braden R. Hale, Bonnie R. Tran, Anne G. Thomas, Michael P. Grillo, Marni B. Jacobs, Jennifer McAnany, Richard A. Shaffer

**Affiliations:** 1 Department of Defense HIV/AIDS Prevention Program, Naval Health Research Center, San Diego, California, United States of America; 2 US Military HIV Research Program, Walter Reed Army Institute of Research, Silver Spring, Maryland, United States of America; 3 Leidos, Inc., Reston, Virginia, United States of America; 4 University of California, San Diego, La Jolla, California, United States of America; South Texas Veterans Health Care System, UNITED STATES

## Abstract

HIV rapid diagnostic tests (RDTs) combined in an algorithm are the current standard for HIV diagnosis in many sub-Saharan African countries, and extensive laboratory testing has confirmed HIV RDTs have excellent sensitivity and specificity. However, false-positive RDT algorithm results have been reported due to a variety of factors, such as suboptimal quality assurance procedures and inaccurate interpretation of results. We conducted HIV serosurveys in seven sub-Saharan African military populations and recorded the frequency of personnel self-reporting HIV positivity, but subsequently testing HIV-negative during the serosurvey. The frequency of individuals who reported they were HIV-positive but subsequently tested HIV-negative using RDT algorithms ranged from 3.3 to 91.1%, suggesting significant rates of prior false-positive HIV RDT algorithm results, which should be confirmed using biological testing across time in future studies. Simple measures could substantially reduce false-positive results, such as greater adherence to quality assurance guidelines and prevalence-specific HIV testing algorithms as described in the World Health Organization’s HIV testing guidelines. Other measures to improve RDT algorithm specificity include classifying individuals with weakly positive test lines as HIV indeterminate and retesting. While expansion of HIV testing in resource-limited countries is critical to identifying HIV-infected individuals for appropriate care and treatment, careful attention to potential causes of false HIV-positive results are needed to prevent the significant medical, psychological, and fiscal costs resulting from individuals receiving a false-positive HIV diagnosis.

## Introduction

The standard method of HIV testing in many developing countries is a simple and inexpensive rapid diagnostic test (RDT) algorithm, as recommended by the World Health Organization (WHO) for diagnosing HIV [1]. RDTs have been validated under various circumstances and, in research study conditions, have near-equivalent sensitivity and specificity to enzyme-linked immunosorbent assay/western blot strategies [2,3]. Under less optimal circumstances, especially in low HIV prevalent regions, the positive predictive value of individual RDTs and RDT algorithms can be low [4–6], thus increasing the number of individuals who may be incorrectly diagnosed as HIV-positive.

**Fig 1:**
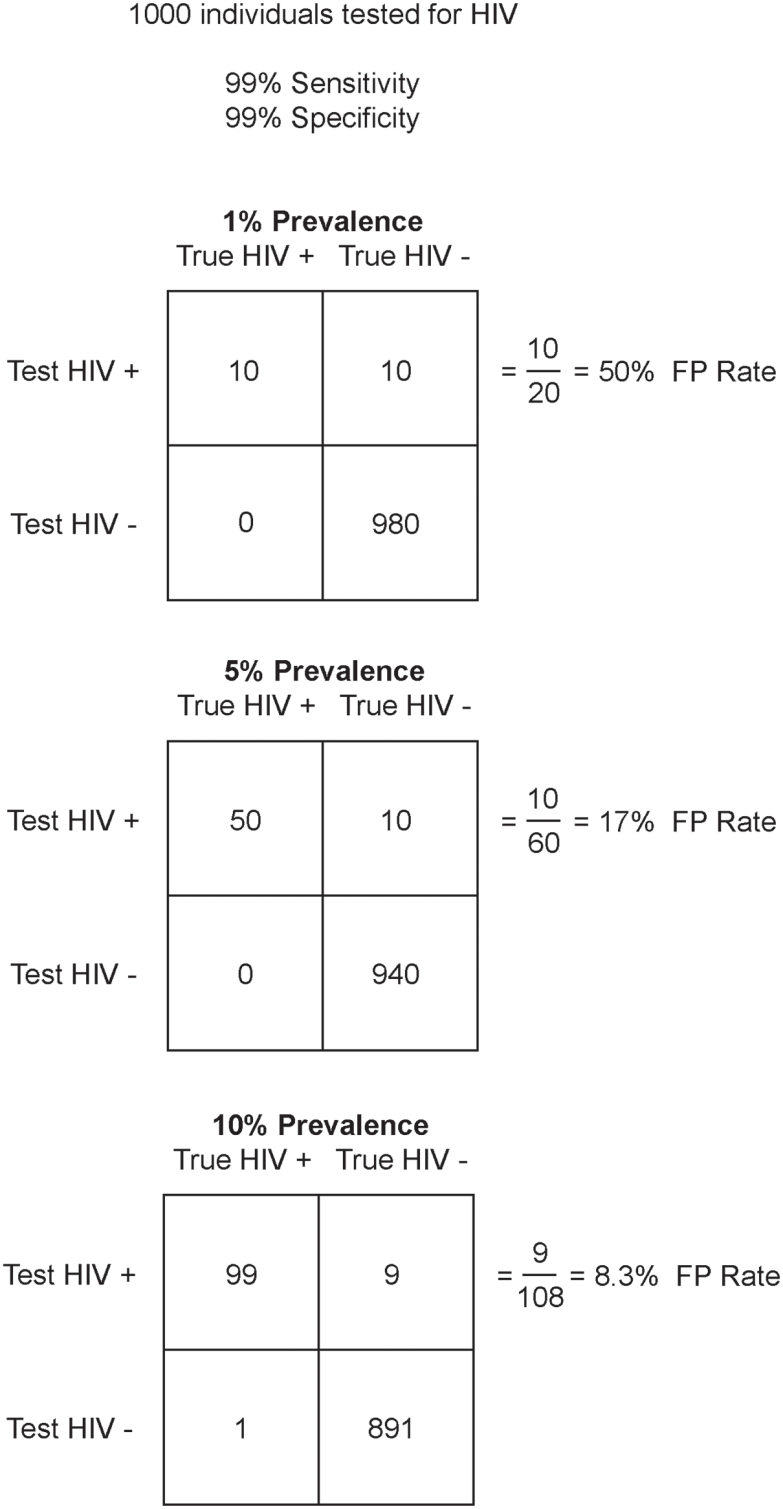
Demonstration of the inverse relationship between HIV prevalence and proportion of individuals who will incorrectly test positive for HIV, using a hypothetical sample size of 1000 individuals and a rapid test with 99% sensitivity and specificity.

Circumstances recognized to increase the likelihood of obtaining a false-positive test result using RDT algorithms include user and clerical errors among staff, over-interpretation of weakly positive test lines, suboptimal testing algorithms or adherence to them, and presence of conditions resulting in polyclonal gammopathy and subsequent cross-reactive antibody reactions, among other factors [1,4,7–10]. The proportion of false-positive tests will increase in low-prevalence countries because the positive predictive value of the test decreases in a mathematically predictable fashion (Fig 1). For this reason, HIV testing guidelines by WHO and the Joint United Nations Programme on HIV/AIDS recommend only diagnosing an individual as HIV-positive after three sequential HIV-positive test results in settings where the HIV prevalence is <5% [11,12]. The degree of adherence to these guidelines in low prevalence settings is unclear, however, several national HIV testing strategies differ from the WHO guidelines and may reduce diagnostic accuracy. After reviewing the results of studies examining false-positive test results, the WHO was prompted to issue a note in October 2014 reinforcing that HIV-positive individuals should be re-tested before initiation of care and/or antiretroviral therapy (ART) [13,14].

RDTs typically demonstrate high sensitivity and high specificity. However, under optimal conditions the first test conducted should be the most sensitive of those available to identify as many HIV-positive individuals as possible, and the second test should have higher specificity to exclude those who are actually HIV-negative [1,11]. This method also relies on the principle that RDTs should contain different HIV antigens to reduce the chance of non-specific cross-reacting antibodies causing a false positive reaction on both rapid tests [1]. Unfortunately, RDT manufacturers have a limited selection of antigens to include in their rapid test kits [4,15], and the RDT antigen composition is proprietary. These factors present challenges to constructing optimal testing algorithms.

When incorrect HIV testing results are reported to individuals based on inaccurate rapid testing, severe consequences may ensue. An investigation conducted in the Democratic Republic of the Congo, Burundi, and Ethiopia examined individuals with misclassified HIV results: an HIV-negative infant who was given antiretroviral prophylaxis; an HIV-negative woman who had ceased to breastfeed upon being told that she was HIV-positive; several HIV-negative adults who had been taking ART for a mean duration of 12.5 months; an HIV-negative woman who had been having unprotected sex with her HIV-positive husband for three years; an HIV-negative woman who married an HIV-positive man after her HIV-negative husband divorced her; and an HIV-negative man whose wife had left him when he told her he was HIV-positive [7].

Data from seven military, cross-sectional HIV prevalence studies utilizing RDT algorithms are presented here, comparing self-reported HIV status to subsequent HIV serotesting results.

## Methods

Data included in the present study were drawn from seven cross-sectional HIV seroprevalence studies in seven sub-Saharan African military populations between 2006 and 2015.

### Human subjects protection and ethical considerations

All study protocols were approved by institutional review boards or human subjects protection organizations at the Naval Health Research Center or Research Triangle International and/or in each partner country. Both male and, where available, female, active duty military personnel were selected for participation using random, convenience, or stratified sampling of the military force. Selected individuals were provided with written and verbal informed consent instructions. Consent was required for inclusion in the study, but written consent utilized a checkbox instead of a signature to protect the participants’ anonymity; this method was approved by human subject protection organizations. Consent was recorded either on a paper or computer-based consent form.

### Survey tool and HIV rapid testing

For all seven partner militaries, a 60- to 90-minute questionnaire was administered, followed by a blood sample collection used for the HIV RDT algorithm consistent with national testing guidelines (serial rapid tests conducted by trained counseling and testing staff). Testing was supervised by study staff to ensure the algorithm was performed correctly. Quality assurance (QA) procedures varied between countries, to include retesting of all positive samples in 5 countries, all indeterminate samples in 6 countries, and variable retesting of HIV-negatives. In one country, we did not have data on a QA process. The survey used for all seven partner militaries was based on a standardized modular survey, which assessed HIV risk behaviors and was developed from previous military surveys and Demographic and Health Surveys. Although standardized, some survey questions were modified to ensure that content was consistent with the local language and culture of the partner military. Participants reported demographic and sexual behavior data through either a self-administered or interview-administered survey (with paper or computer-based methods).

### Questions used to assess self-reported HIV status

Questions used to assess previous HIV testing status varied slightly by country. For Military A, participants were asked to provide responses to two sequential questions: (1) “Have you ever been tested for HIV?” (response options: yes; no; don’t know); and (2) “What were the results of your most recent HIV test?” (response options: HIV-positive; HIV-negative; I did not receive the results; prefer not to answer). Those who answered “yes” or “don’t know” to the first question and provided an HIV status to the second question were included in the analysis. For Militaries B and D, participants were asked to provide responses to three sequential questions: (1) “Prior to coming here today, have you ever been tested to see if you have the AIDS virus?” (response options: yes; no); (2) “If yes, did you get the results of the test?” (response options: yes; no; not applicable, never been tested); and (3) “If yes, was your HIV status positive or negative?” (response options: negative; positive; not applicable, never been tested; not applicable, never received test results; don’t know). Those who answered “yes” to both the first and second questions and provided a response to the third question were included in the analysis. For remaining militaries, participants were instructed to provide responses to two sequential questions: (1) “The last time you had sexual intercourse, did you know your own HIV status?” (response options: yes; no; not applicable [Military C, F]; yes; no; prefer not to answer; not applicable, I have never had sexual intercourse [Military E, G]); and (2) “Was your HIV status positive or negative?” (response options: positive; negative; not applicable [Military C, F]; positive; negative; decline to provide HIV status [Military E]; positive; negative; prefer not to answer [Military G]). All participants who provided a response to the second testing question (regardless of whether they answered the first question affirmatively) were included in the analysis.

### Analysis

HIV test results were cross-tabulated to the survey responses for analysis. The proportion of participants who reported their HIV status as HIV-positive but subsequently tested HIV-negative was reported. To preserve confidentiality, the exact prevalence of HIV was not reported. Proportion of missing data was calculated, including the number of individuals who did not or refused to answer the question in the numerator divided by the number of total survey participants.

Data were analyzed using SAS software, version 9.4 (SAS Institute, Inc., Cary, North Carolina). Three separate multivariable logistic regression models were conducted to examine whether demographics (age, sex, education, marital status) and knowledge indicators (United Nations General Assembly Special Session on HIV/AIDS, UNGASS) were significantly associated with: (model 1) self-reported HIV-positive status, (model 2) a self-reported HIV status that was discordant with the RDT algorithm result, or (model 3) testing HIV-positive using the RDT algorithm. Participants were classified as being knowledgeable about HIV transmission prevention if they answered all five UNGASS questions correctly (i.e., transmission is reduced by having sex with one faithful uninfected partner and using condoms, healthy-looking individuals can have HIV and HIV cannot be transmitted from mosquito bites or sharing a meal with an HIV-infected individual). The first regression model included participants with an HIV-negative RDT algorithm result and the second model included all participants (those with both HIV-negative or HIV-positive RDT algorithm result); both models assessed the probability of the participant self-reporting their HIV status as discordant with their actual HIV RDT algorithm result. The third model included all participants and assessed the probability of testing HIV-positive using the RDT algorithms. All p-values were based on two-tailed tests of significance, defined as p<0.05.

## Results

The proportion of participants who reported they were HIV-positive but subsequently tested HIV-negative ranged from 3.3% to 91.1% among the seven militaries (Table 1). The proportion of participants who did not provide a response to the survey questions used in the analysis ranged from 3.9% to 19%, with the majority (71%, *n* = 5/7 countries) as ≤ 10% missing. Militaries A and B had the largest proportion of individuals who reported they were HIV-positive but subsequently tested HIV-negative using a standardized RDT algorithm after completion of the survey. For Military A, among the 146 participants who reported they were HIV-positive on their last HIV test, 133 (91.1%) tested negative for HIV. For Military B, among the 30 participants who reported they were HIV-positive, 12 of these participants (40%) tested negative for HIV. Of the remaining militaries, over 10% of the participants in Militaries C and D (13.5% and 17.2%, respectively) reported they were HIV-positive but tested HIV-negative. Militaries E, F, and G had the smallest number of HIV-negative participants who self-reported they were HIV-positive (8.3%, 6.3%, and 3.3%, respectively). Individuals who perceived they were at low risk for HIV infection tested HIV-negative more often than individuals who perceived they were at high risk (proportion of HIV-negative tests among those perceiving low risk vs. high risk: Military C: 75.8% vs. 68.1%; Military E: 91.2% vs. 79.7%; Military F: 90.3% vs. 58.0%), although no statistical comparison was conducted.

**Table 1:** Military-specific prevalence of testing HIV-positive using the rapid diagnostic test algorithm among those who self-reported being HIV-positive.

Self-reported HIV-positive status	Total	Tested HIV-positive			Tested HIV-negative			HIV Prevalence^e^
	*N*	*N*	(%)^a^	95% CI	*n*	(%)^a^	95% CI	(%)
Military A^b^	146	13	(8.9)	5.0–15.0	133	(91.1)	84.9–94.9	≤ 5
Military B^c^	30	18	(60.0)	40.7–76.8	12	(40.0)	23.2–59.3	≤ 5
Military C^d^	29	24	(82.8)	63.5–93.5	5	(17.2)	6.5–36.5	≥ 15
Military D^c^	96	83	(86.5)	77.6–92.3	13	(13.5)	7.7–22.4	10–14
Military E^d^	36	33	(91.7)	76.4–97.8	3	(8.3)	2.2–23.6	≥ 15
Military F^d^	32	30	(93.8)	77.8–98.9	2	(6.3)	1.1–22.2	≥ 15
Military G^d^	90	87	(96.7)	89.8–99.1	3	(3.3)	0.9–10.1	≥ 15

^a^Percentage of sample who self-reported HIV positivity and then subsequently tested HIV-positive or HIV-negative, by row.

^b^Response based on the following questions: Have you ever been tested for HIV? What were the results of your most recent HIV test?

^c^Response based on the following questions: Prior to coming here today, have you ever been tested to see if you have the AIDS virus? If yes, did you get the results of the test? If yes, was your HIV status positive or negative?

^d^Response based on the following questions: The last time you had sexual intercourse did you know your own HIV status? Was your HIV status positive or negative?

^e^HIV prevalence ranges reported were calculated based on the total number of individuals with an HIV RDT algorithm positive test result divided by all individuals who received an HIV RDT as part of each country study.

Among participants whose RDT algorithm test result was HIV-negative, after adjusting for age, sex, education, marital status and number of UNGASS questions answered correctly, those who were 40 years or older (OR 3.29, 95% CI, 1.83–5.91), completed a primary education (OR 5.83, 95% CI, 2.59–13.10), were single (OR 2.22, 95% CI, 1.56–3.17) or incorrectly answered one or more UNGASS question (OR 3.95, 95% CI, 2.66–5.88) were significantly more likely to self-report their HIV status as positive than those 18–24 years, college educated, married/ living together and those who answered all 5 UNGASS questions correctly (Table 2, Model 1).

**Table 2:** Adjusted odds of demographic and knowledge factors associated with incorrectly self-reporting HIV positive status (Model 1), incorrectly self-reporting HIV status (Model 2), and testing HIV positive using the RDT algorithm (Model 3).

	Model 1^a^	Model 2^b^	Model 3^c^
	(n = 4257)	(n = 4607)	(n = 4607)
Characteristic	Adjusted OR (95% CI)	Adjusted OR (95% CI)	Adjusted OR (95% CI)
Age, years			
18–24	1.00	1.00	1.00
25–29	0.98 (0.50–1.91)	1.86 (1.12–3.09)	5.77 (2.64–12.62)
30–34	1.55 (0.80–3.00)	2.30 (1.35–3.92)	11.82 (5.44–25.69)
35–39	2.77 (1.49–5.13)	3.35 (1.99–5.62)	21.22 (9.78–46.06)
40+	3.29 (1.83–5.91)	3.70 (2.26–6.07)	26.57 (12.38–57.02)
Sex			
Male	1.16 (0.35–3.89)	0.50 (0.26–0.94)	0.25 (0.15–0.44)
Female	1.00	1.00	1.00
Education			
Did not attend school	10.49 (3.50–37.80)	8.42 (3.43–20.66)	1.20 (0.35–4.17)
Primary or less	5.83 (2.59–13.10)	4.12 (2.31–7.36)	1.54 (0.98–2.41)
Secondary	2.13 (0.94–4.79)	2.16 (1.23–3.80)	2.40 (1.64–3.51)
High school	1.5 (0.64–3.47)	1.72 (0.96–3.07)	0.96 (0.63–1.46)
College/University/Technical	1.00	1.00	1.00
Marital status			
Single	2.22 (1.56–3.17)	1.70 (1.29–2.23)	0.87 (0.67–1.13)
Married/Living together	1.00	1.00	1.00
UNGASS			
All 5 questions correct	1.00	1.00	1.00
Missed ≥ 1 question	3.95 (2.66–5.88)	2.79 (2.11–3.69)	1.03 (0.81–1.30)

^a^Model 1 = Among participants with an HIV negative RDT algorithm result, modeling the probability of self-reporting HIV positive status

^b^Model 2 = Among all participants with an HIV RDT algorithm result, modeling the probability of incorrectly self-reporting HIV status

^c^Model 3 = Among all participants with an HIV RDT algorithm result, modeling the probability of having an HIV positive RDT algorithm result

Similarly, among all participants (incorporating those with both positive and negative RDT algorithm results), after adjusting for the same variables listed above and using the same reference categories, participants who were 40 years or older (OR 3.70, 95% CI, 2.26–6.07), completed a primary education (OR 4.12, 95% CI, 2.31–7.36), were single (OR 1.70, 95% CI, 1.29–2.23), or incorrectly answered one or more UNGASS question (OR 2.79, 95% CI 2.11–3.69) were significantly more likely to report their HIV status in discordance with their RDT algorithm result (Table 2, Model 2). Males were significantly less likely to report their HIV status in discordance with their RDT algorithm result (OR 0.50, 95% CI, 0.26–0.94) than females.

Among all participants (incorporating those with both positive and negative RDT algorithm results), after adjusting for all variables, older age, secondary education and female sex were also significantly associated with having an HIV-positive RDT algorithm result, but marital status and incorrectly answering the UNGASS questions were not significantly associated (Table 2, Model 3).

## Discussion

Discordance between HIV-negative RDT algorithm results and participants who self-reported their HIV status as positive ranged from 3.3% to 91.1% among the seven military populations. The lowest HIV prevalence populations had the highest proportion of individuals who self-reported HIV-positive status but subsequently tested HIV-negative. The rates from these analyses are consistent with a decreased positive predictive value in the lower-prevalence settings (Fig 1). There is a predictable correlation between disease prevalence and false-positive test results even when test performance is unchanged. The WHO RDT algorithm recommended for use in low prevalence countries should yield excellent sensitivity and specificity but many of these countries are utilizing suboptimal RDT algorithms and/or procedures. The discovery of a significant fraction of people who believe they are HIV positive but are actually HIV-negative has significant implications for future programming.

For low HIV prevalence settings, WHO recommends that three RDTs should be employed in RDT algorithms [1,11], but our understanding is that RDT cost and supply chain issues may limit the ability for these countries to follow such recommendations. However, following WHO guidelines and optimizing other RDT procedures have been shown to improve testing accuracy. It was previously reported that after an improved testing algorithm was introduced in Burundi, which required an additional confirmatory test for those who tested positive and additional modifications to the testing algorithm, the false-positive rate dropped to zero [7].

### Technical considerations

Technical limitations have been shown to increase false-positive HIV test results in voluntary counseling and testing centers lacking rigorous standardized operating procedures among staff in high stress and workload environments, and in settings with insufficiently trained staff [7,9]. Misinterpretation of weakly positive test lines as HIV-positive, in conjunction with algorithms that include tiebreaker tests which are not recommended [1], has been shown to result in a clinically unacceptable number of false-positive algorithm results [4,16,17]. Although reclassification of weakly positive tests as negative may affect sensitivity, the impact is often quite low [4]. Categorizing only individuals with two strongly positive RDTs as a positive result reduced false-positive rates by 69% and 95%, in the Democratic Republic of Congo and Uganda, respectively, without a significant change in test sensitivity [16,18]. In programmatic settings, treating a weakly positive test line as an indeterminate result and re-testing by an enzyme immunoassay and western blot (or equivalent) is advised [16].

### Intrinsic test limitations

Cross-reactive antibodies from polyclonal immune activation among individuals with other diseases can result in false-positive RDTs. For example, Lejon et al. used eight common RDTs in patients infected with *Trypanosoma brucei gambiense* (African trypanosomiasis) and reported HIV RDT false-positive rates between 35% and 95% [6]. This issue is not exclusive to RDTs; for example, Everett et al. reported a high HIV false-positive test rate (92%) among adolescents with active schistosomiasis in Tanzania using 4^th^ generation EIA [19,20]. In addition, different RDTs may employ identical target antigens, due to a limited number of HIV antigens for RDTs [4]. Variation of RDT antigens allows for more accurate RDT algorithms in the setting of cross-reactive antibodies.

### Study limitations

Participants may have incorrectly reported previous HIV test results, since this information was based on self-report. However, questions used to assess previous HIV test results were very specific. Also, it seems unlikely that most individuals would misremember a previous HIV-positive test result given the personal impact of the diagnosis. However, participants who incorrectly answered one or more UNGASS questions were significantly more likely to report their HIV status in discordance with their HIV test result, indicating limited HIV transmission knowledge. Although several demographic factors (age, education, sex) also were associated with actually testing HIV positive using the RDT algorithm, incorrect UNGASS questions were not. A lower HIV literacy associated with discordant self-reported HIV testing and current results suggests some of the effect may be due to improper understanding of “HIV-positive”; however, the small effect size suggests this is not likely to account for much of our findings. Future studies could compare sequential biological HIV testing to confirm results reported in this study. Anecdotal reports from sub-Saharan African populations suggest some individuals become convinced they are HIV-positive due to sexual risk-taking behavior, even in the absence of HIV testing. Limited data examining this phenomenon are inconclusive [21]. This scenario seems an unlikely explanation of these findings, given post-hoc analyses showed the expected association between perception of risk and HIV status. Individuals who reported they were not at all likely to acquire HIV tested HIV-negative more often than individuals who reported a high probability of acquiring HIV. This suggests the individual’s perception of risk was in concordance with their actual infection status.

We also considered the possibility that subjects could interpret “HIV-positive” to mean a “good” test result (and presuming a good or desirable result is actually HIV-negative). Anecdotally, we have found that understanding of the term “HIV-positive” is quite high in most African countries. The presence of substantial variation in the results between countries also argues against this misinterpretation, since the question did not vary greatly between surveys. Translation issues or local understanding could play a role here, but translation of “HIV-positive” is not generally difficult.

The study results could also be due to subject entry error or misunderstanding of study questions. However, the survey was generally administered through randomized sampling methods in a similar fashion across all seven countries. There may be some bias introduced which may have some unknown impact on the results introduced by the few sites collected through convenience sampling, but we assess the impact to be limited. The modules were very similar, with the exception of local preference in phrasing and translation. Because the surveys were similar, we think the differences in the proportion of individuals with an HIV result discordant with their self-reported HIV status between countries is more likely due to prevalence and systemic errors rather than survey error rate. Proportion of missing responses to the survey questions used in the analysis varied by country, which may have also under or overestimated the proportion of presumed false-positive test results, and should be validated in future studies. We also assumed that the self-report of HIV positivity was incorrect if the current RDT algorithm was negative; theoretically, the prior test result could be correct. However, the current RDT algorithm testing was performed according to country-specific national testing guidelines, whereas we are unable to determine the quality of prior testing; therefore, we think if there was erroneous testing, it is more plausible that the prior test results were incorrect. In addition, quality assurance and quality control of algorithm results was done in accordance with national policy, but there was significant variation in these policies.

Not all surveys assessed information regarding whether people already identified as HIV-positive were taking ART so this was not examined in the current analysis. Previous research suggests that RDT sensitivity may decline in the setting of long term ART [22]. This is a limitation of the study data; however, we believe this effect to be a cumulatively small effect under the study circumstances and unlikely to result in the study findings.

It is possible that test results could vary based on which RDT algorithm was used in each country. Each country used the RDT algorithm consistent with the national standard and therefore differed by country. Additional research would be needed to confirm whether or not this factor influenced reported findings. Within the study, the participant provided information on their last HIV test, without referencing any specific time frame. If the original test and the study RDT algorithm were conducted within a short time period, the assumption was the country algorithm would not have changed during this time. However, it is possible if a substantial period of time had passed between the initial test result and the study conducted test result, country RDT algorithms and RDT vendors may have changed during that time. Given that RDT sensitivities and specificities across tests are very high, it is unlikely an individual’s RDT algorithm test result would vary across time based on a different RDT vendor, unless the algorithm or standard operating procedures were suboptimal.

## Conclusion

This study suggests false-positive RDT algorithm results remain a significant problem, with varying implications for our participants. There is a need for validation in future studies to confirm RDT algorithm result records of the previous and subsequent test algorithm results. Fortunately, simple improvements in algorithms, RDT interpretation, and re-evaluation of indeterminate test results have been shown to reduce false-positive HIV rates. Careful attention and consideration should be given to managing HIV testing using RDT algorithms in low-prevalence countries in order to optimize reduction of false-positive RDT algorithm results. Additional studies are needed to confirm results reported in this manuscript by calculating false-positive rates based on biological HIV algorithm test results across time.
